# Fabrication and Evaluation of a Novel Non-Invasive Stretchable and Wearable Respiratory Rate Sensor Based on Silver Nanoparticles Using Inkjet Printing Technology

**DOI:** 10.3390/polym11091518

**Published:** 2019-09-18

**Authors:** Ala’aldeen Al-Halhouli, Loiy Al-Ghussain, Saleem El Bouri, Haipeng Liu, Dingchang Zheng

**Affiliations:** 1 Mechatronics Engineering Department/NanoLab, School of Applied Technical Sciences, German Jordanian University, P.O. Box 35247, Amman 11180, Jordan; loui.essam@hotmail.com (L.A.-G.); saleem.bouri@hotmail.com (S.E.B.); 2 Institute of Microtechnology, Technische Universität Braunschweig, 38124 Braunschweig, Germany; 3 Faculty of Engineering, Middle East University, Amman 11180, Jordan; 4 Mechanical Engineering Department, University of Kentucky, Lexington, KY 40506, USA; 5 Medical Device and Technology Research Laboratory, School of Allied Health, Faculty of Health, Education, Medicine and Social Care, Anglia Ruskin University, Chelmsford CM1 1SQ, UK; haipeng.liu@anglia.ac.uk (H.L.); dingchang.zheng@anglia.ac.uk (D.Z.)

**Keywords:** respiratory rate, wearable sensors, stretchable circuits, inkjet printing, PDMS

## Abstract

The respiration rate (RR) is a key vital sign that links to adverse clinical outcomes and has various important uses. However, RR signals have been neglected in many clinical practices for several reasons and it is still difficult to develop low-cost RR sensors for accurate, automated, and continuous measurement. This study aims to fabricate, develop and evaluate a novel stretchable and wearable RR sensor that is low-cost and easy to use. The sensor is fabricated using the soft lithography technique of polydimethylsiloxane substrates (PDMS) for the stretchable sensor body and inkjet printing technology for creating the conductive circuit by depositing the silver nanoparticles on top of the PDMS substrates. The inkjet-printed (IJP) PDMS-based sensor was developed to detect the inductance fluctuations caused by respiratory volumetric changes. The output signal was processed in a Wheatstone bridge circuit to derive the RR. Six different patterns for a IJP PDMS-based sensor were carefully designed and tested. Their sustainability (maximum strain during measurement) and durability (the ability to go bear axial cyclic strains) were investigated and compared on an automated mechanical stretcher. Their repeatability (output of the sensor in repeated tests under identical condition) and reproducibility (output of different sensors with the same design under identical condition) were investigated using a respiratory simulator. The selected optimal design pattern from the simulator evaluation was used in the fabrication of the IJP PDMS-based sensor where the accuracy was inspected by attaching it to 37 healthy human subjects (aged between 19 and 34 years, seven females) and compared with the reference values from e-Health nasal sensor. Only one design survived the inspection procedures where design #6 (array consists of two horseshoe lines) indicated the best sustainability and durability, and went through the repeatability and reproducibility tests. Based on the best pattern, the developed sensor accurately measured the simulated RR with an error rate of 0.46 ± 0.66 beats per minute (BPM, mean ± SD). On human subjects, the IJP PDMS-based sensor and the reference e-Health sensor showed the same RR value, without any observable differences. The performance of the sensor was accurate with no apparent error compared with the reference sensor. Considering its low cost, good mechanical property, simplicity, and accuracy, the IJP PDMS-based sensor is a promising technique for continuous and wearable RR monitoring, especially under low-resource conditions.

## 1. Introduction

The respiration rate (RR) is a vital sign that is a clinical indication of many cardiorespiratory diseases such as cardiac arrest, infections, and congenital respiratory disorders [1]. Despite its clinical significance, currently, clinical RR measurement mainly depends on manual recording or an expensive apparatus, which is either inaccurate or inappropriate for continuous RR monitoring under low-resource conditions. On the other hand, accurate continuous RR monitoring is difficult to achieve due to the lack of accurate, convenient, low-cost, and user-friendly sensors. There is an urgent need for accurate, portable, convenient, and low-cost sensors for continuous RR monitoring.

Wearable flexible sensors (WFSs) provide a probable solution for continuous RR monitoring [2,3,4,5,6]. WFSs detect RR from chest wall motion through two mainstream methods: impedance measurement and respiratory inductance plethysmography (RIP) [2,7,8,9,10]. For instance, Lee [3] developed a piezoelectric sensor for RR measurements while Ciocchetti et al. [5] developed a smart textile sensor to measure RR based on the differential motion of the abdomen and the chest during respiration. Moreover, Hesse et al. [11] developed a chest-strap sensor for respiration monitoring using a force-sensing resistor, which was compared with an ergo-spirometry system as a reference. They reported an error of −0.32 ± 0.68 Hz (Mean difference ± Standard Deviation of difference) compared with the reference sensor. However, these sensors are burdensome with electromagnetic radiation and high energy consumption, and inconvenient for daily use, especially under low-resource conditions, with the worldwide concern on global warming [12] and increase in energy demand forcing industries to continuously look for enhancements to their processes and energy usage [13,14].

Stretchable circuits play an important role in fulfilling more convenient wearable sensors [15,16,17,18,19,20,21,22]. In different applications, the fabrication of stretchable circuits involves the use of various deposition techniques such as photolithography [6,23], screen printing [24,25,26], and inkjet printing [27,28,29,30,31]. There are three studies on the fabrication and development of stretchable wearable sensors for RR measurement [6,32,33] using lithographic technologies [6,32] and other complicated techniques [33] with multilayer configuration. The developed sensor in reference [6] had skin-like surface properties with resistance to deformation, but the use of a femtosecond laser and sophisticated lithographic process complicated the fabrication [6]. Similar to reference [6], the studies from Chung et al. [32] and Chu et al. [33] used sophisticated techniques for the development of the RR stretchable sensor. With a lower fabrication cost, and fewer processing steps than photolithography and screen printing, inkjet printing is a promising technology for the fabrication of skin-like wearable RR sensors.

The studies in the literature presented several stretchable and wearable sensors for continuous RR monitoring but they are expensive [6] or low-cost but insufficient in stretchability [2,11] or wearability [34]. Therefore, this study aimed to develop and evaluate a low-cost stretchable and wearable sensor for continuous RR monitoring based on inkjet printing on the substrates of Polydimethylsiloxane (PDMS).

## 2. Materials and Methods

**Figure 1:**
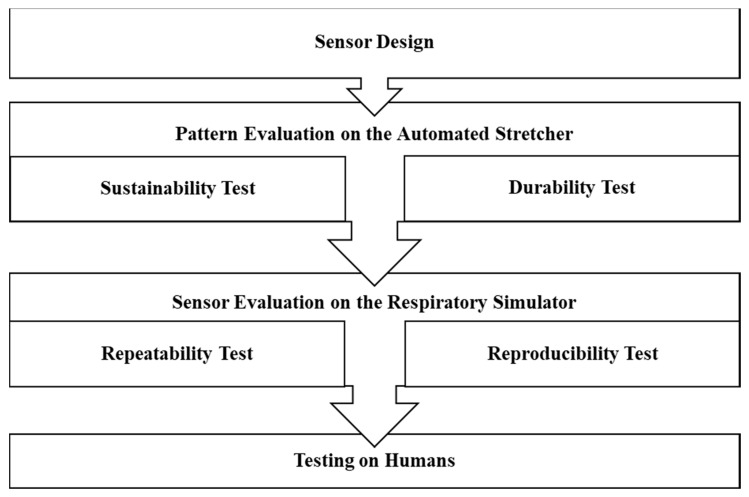
Development procedure for the fabrication and evaluation of the respiration rate (RR) sensor.

The development of the inkjet-printed (IJP) PDMS-based sensor consisted of four major stages. Firstly, the IJP PDMS-based sensor was designed and fabricated with six different design patterns. Secondly, the sustainability and durability of the IJP PDMS-based sensors were investigated on an automated stretcher to select the best pattern among the six designed patterns. Thirdly, the IJP PDMS-based sensor with the best pattern was tested on the respiratory simulator to evaluate its repeatability (output of the same sensor from repeated tests at identical conditions) and reproducibility (output from different sensors with the same design at identical conditions). Finally, the validation of IJP PDMS-based sensor was performed on healthy human subjects. Figure 1 shows the development procedure in this study for the fabrication and evaluation of the RR sensor.

### 2.1. Materials and Equipment

#### 2.1.1. Processing of PDMS Substrates

PDMS is a silicon-based elastomer that is widely used for the fabrication of stretchable circuits since it is durable, commercially available, and compatible with biomedical applications and human bodies [23,24,35,36,37]. Transparent PDMS substrates were prepared using Dow Corning^®^ Sylgard 184 (Midland, MI, USA). Base PDMS material was mixed with the curing agent in a volume ratio of 10 to 1 as recommended [16,38]. The mixture was poured into 100 mm × 40 mm rectangular acrylic molds. For each mold, 4 mL PDMS mixture was used to produce a PDMS substrate with 1mm thickness. The molds were then placed in the vacuum oven at room temperature for 30 min for degasification. After that, the oven temperature was kept at 70 °C for 2 h for PDMS curation [39,40].

The derived PDMS surfaces were hydrophobic, so proper physical or chemical treatment was necessary to enable the surface to formulate the conductive patterns. To enhance the hydrophilicity, the PDMS surfaces were treated with ZEPTO Diener etcher (Diener electronic GmbH, Ebhausen, Germany) using plasma barrel etching technology. PDMS surfaces were processed at full power (50 Watt) in the plasma etcher for 15 min. The hydrophilicity of the surface was enhanced by the reaction with the processed gas (O_2_ in this study). The reaction breaks the material’s surface to volatile and small molecules, which were removed by a vacuum pump [41].

#### 2.1.2. Inkjet Printing

**Figure 2:**
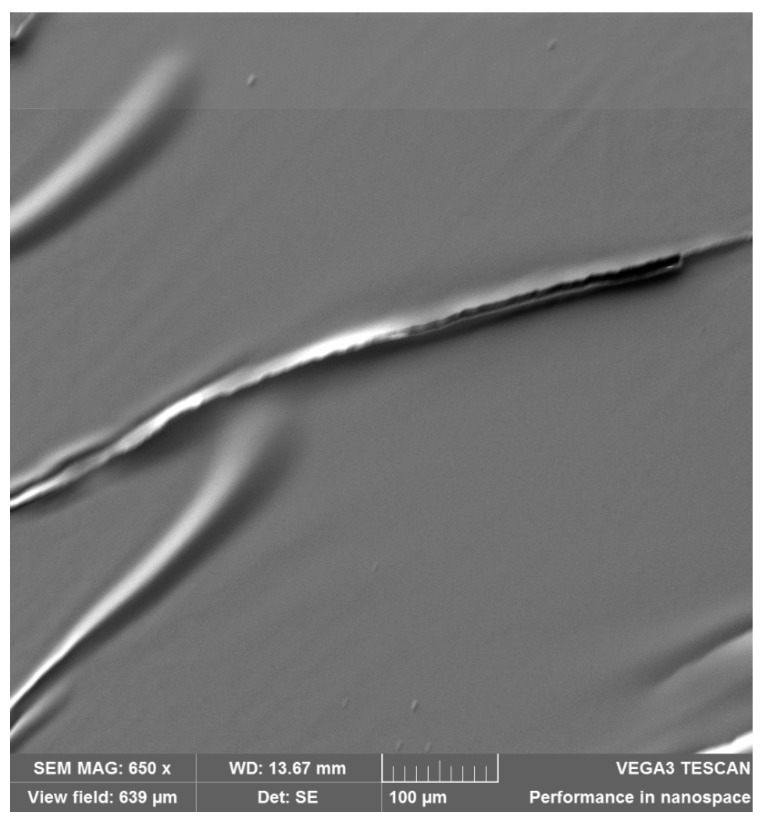
Scanning electron microscope (SEM) microscopic image of the dispersed silver nanoparticles on the Polydimethylsiloxane (PDMS)substrate.

The conductive silver nanoparticles ink (Silverjet DGP-40LT-15C, Sigma-Aldrich, Inc., St. Louis, MO, USA) was precisely injected on the treated PDMS substrates. The ink consists of a solvent (30–35 wt % dispersion in triethylene glycol monomethyl ether) with suspended Silver Nanoparticles (NPs). This ink is chemically stable with high electrical conductivity [42]. The injection was performed with an inkjet printer (Fujifilm Dimatix Material Printer DMP-2831, FUJIFILM Dimatix, Inc., Santa Clara, CA, USA) in drop on demand mode. The inkjet printer can print in a resolution down to 20 µm using the 1 pL cartridge printhead, and down to 125 µm with the 10 pL printhead [43]. Two print heads (1 and 10 pL) were used in this study to fulfil the different design patterns with different resolutions (refer to Section 2.1.3). The filled cartridge with silver nanoparticles ink was placed on a vortex mixer to ensure that the silver nanoparticles are well dispersed in the ink, which in return will ensure appropriate and uniform dispersion of the silver nanoparticles on the PDMS substrate, Figure 2 shows a microscopic image of the dispersed silver nanoparticles on the PDMS substrate taken by TESCAN VEGA3 Scanning Electronic Microscope (SEM), (Brno, Kohoutovice, Czech Republic).

#### 2.1.3. Design of Conductive Patterns

**Figure 3:**
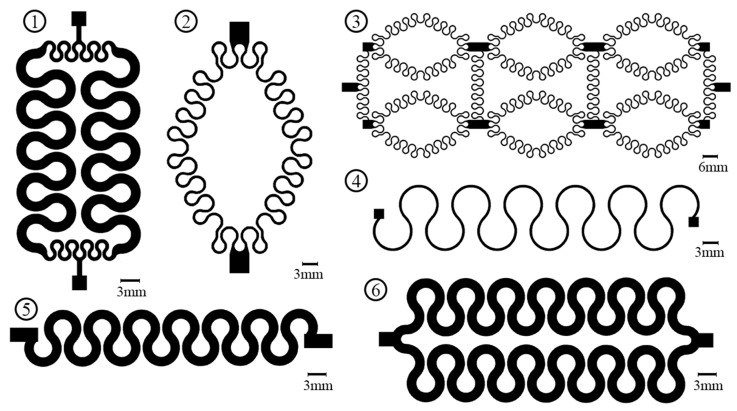
Diagram of the six horseshoe patterns.

The mechanical properties of the IJP PDMS-based sensor depend on the shape of the conductive pattern. Six different patterns were designed, fabricated, and evaluated where the evaluation is explained in details in Section 2.4. The horseshoe pattern sustains large amount of strains as reported in reference [16] that reached up to 25% depending on its dimensions, therefore, all the designs were based on the horseshoe shape. Figure 3 shows the six horseshoe designs based on the horseshoe arrays.

The patterns were designed using AutoCAD (2018, Autodesk, Autodesk, San Rafael, CA, USA) and then exported as dxf files to Eagle (2018, Autodesk, Autodesk, San Rafael, CA, USA). Eagle was used to convert the dxf files into images with a certain resolution. The images were then modified with GNU Image Manipulation (GIMP, Berkeley, CA, USA) and made suitable for the inkjet printer pattern editor.

In inkjet printing, different printheads and drop spacing (DS) were used for different patterns. The 10 pL printhead was used in this study for Patterns 4, 5, and 6. For Patterns 1, 2, and 3, the 1 pL printhead was used due to the small features with 500 and 720 µm line widths and distance between lines of less than 100 µm. For the 10 pL printhead, 30 DS was adopted as in existing studies [16,38]. For the 1 pL printhead, 15 DS was selected after optimization.

### 2.2. Sensor Fabrication

**Figure 4:**
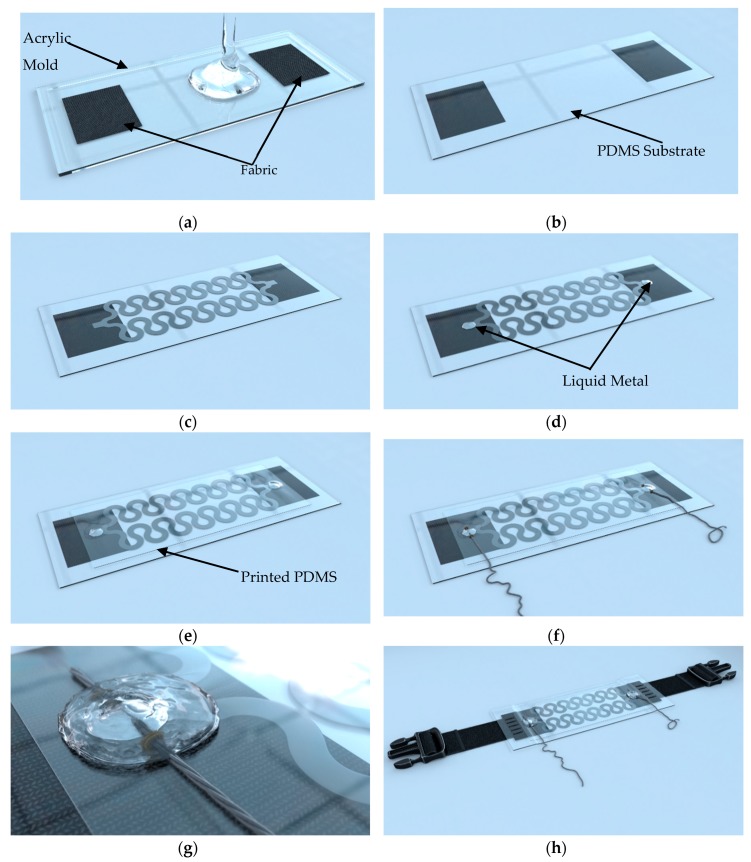
The fabrication process of the respiratory rate sensors: (**a**) pouring the PDMS mixture on the fabric; (**b**) cured PDMS sample with embedded fabrics; (**c**) conductive pattern deposition using the inkjet printer; (**d**) pouring small drops of liquid metal at the pattern’s pads; (**e**) coating the circuit with 10 layers of PDMS; (**f**) sewing the conductive threads over the liquid metal; (**g**) developing PDMS incubators for the liquid metal and (**h**) sewing the sensor to the fabric belt. It should be noted that the presented pattern in the fabrication procedure here is just for demonstration.

The fabrication of the IJP PDMS-based sensor involved several procedures, as summarized in Figure 4. The fabric belt was chosen as the form of the RR sensor attachment mechanism to the human body [11]. A reliable attachment was necessary between the IJP PDMS-based sensor and the belt. Sewing the PDMS on the belt would create cracks in the PDMS and cause the PDMS specimen to collapse. Therefore, small fabric pieces (10 mm × 20 mm) were embedded in the PDMS to enforce the PDMS at the ends and to maintain its stretchability at the same time. The fabric was placed in the acrylic molds before pouring the PDMS mixture, where the procedure (in Section 2.1.1) was then followed to produce the PDMS substrate (Figure 4a,b).

The fabrication of the inkjet-printed circuit was the major process in the fabrication of the IJP PDMS-based sensor (Figure 4c). After the deposition of the silver ink on the PDMS substrate using the inkjet printer, the substrate was then placed in the oven for one hour at 110 °C for sintering. Afterwards, the conductive patterns were coated by ink-jetted PDMS layers to avoid scratches and chemical reactions with air. The PDMS coating layer was prepared by mixing the PDMS with toluene with a volume ratio of 1 to 5 (PDMS: toluene) in order to obtain the viscosity between 10 and 12 cP suitable for the inkjet printer [16]. Before printing the PDMS layers, small quantities of non-hazardous gallium-indium liquid metal (EGaIn, Sunnyvale, CA, USA) were placed on the pads (Figure 4d). The use of the liquid metal could prevent the coverage of the pads by the PDMS while protecting of the remaining parts of the circuit. During the printing process, the platen heating of the inkjet printer was turned on (at 60 °C) in order to allow the sintering of the PDMS coated layer. In this study, ten layers of transparent PDMS were printed on the top of the conductive patterns (Figure 4e).

After the PDMS coating, the sensor was placed in the oven for full PDMS curation for one hour at 110 °C. Afterwards, conductive threads were sewed on the top of the pads with the existence of liquid metal to ensure the conductivity between the conductive patterns and the conductive threads (Figure 4f). Then, PDMS mixture was poured on the liquid metal at the pads in order to create incubation chambers (Figure 4g). Finally, the sensor was sewed from the two fabric ends to a belt to act as the mounting mechanism (Figure 4h).

The deposition of the silver nanoparticles on the PDMS—after proper surface treatment and by using the proper printing parameters (reaching the percolation threshold) [44]— allows the formulation of percolation networks that explains the development of conductive traces by the silver nanoparticles. Applying a force on the stretchable substrate will increase the distance between the silver nanoparticles in and also increases the amount of the tunneling barrier (the air) between the nanoparticles which will increase the resistance of the conductive path [44,45,46] and so decreasing the percolation effect and increasing the tunneling mechanism.

### 2.3. Electronics Implementation for Respiratory Rate Derivation

**Figure 5:**
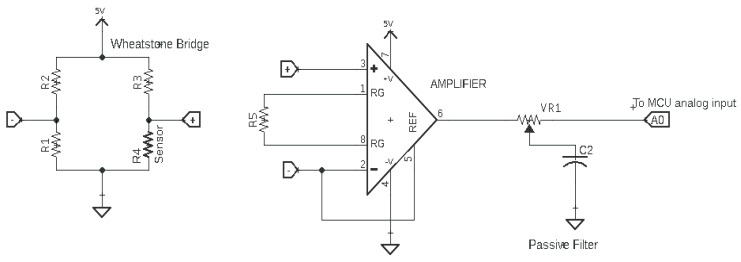
Schematic diagram of a Wheatstone circuit.

The sensor was connected and implemented in a quarter bridge Wheatstone configuration, in which three known resistances were installed on the circuit board, while the fourth resistance was the IJP PDMS-based sensor, note that the supply voltage is equal to five volts, which is supplied from the microcontroller. Figure 5 shows the schematic diagram of a Wheatstone circuit.

**Figure 6:**
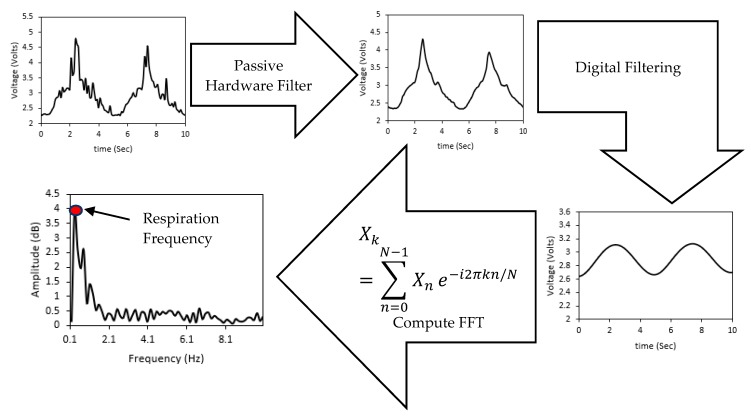
The flow chart of the output signal from the developed sensor.

The voltage difference between the two nodes was calculated in each branch. However, since the difference in voltage is normally in millivolts in the Wheatstone bridge configuration, the two nodes were used as inputs for an instrumentational amplifier to amplify the voltage difference in the bridge circuit with a gain of 100 followed by a passive low-pass filter which was the same for all the evaluated horseshoe patterns. A Matlab Simulink model was developed to determine the RR value by deriving the frequency components of the input signal. Figure 6 shows the process of detecting the RR value, where the Simulink program starts by acquiring the data from the analog circuit at a sampling rate of 100 Hz. And since the acquired data is in the ADC scale, the values are converted to the 5 volts scale and then applied to a third-order Butterworth low-pass filter with a cut-off frequency of 1 Hz [11]. Finally, the filtered signal was processed using Fast Fourier Transform (FFT) block in Simulink and the dominant frequency was analyzed using the spectral analysis block, which plots the frequency spectrum and shows the dominant frequency of the input signal (the respiration rate).

### 2.4. Sensor Validation: Sustainability, Durability, Repeatability, and Reproducibility Tests

#### 2.4.1. Sustainability and Durability Test on the Automated Stretcher

**Figure 7:**
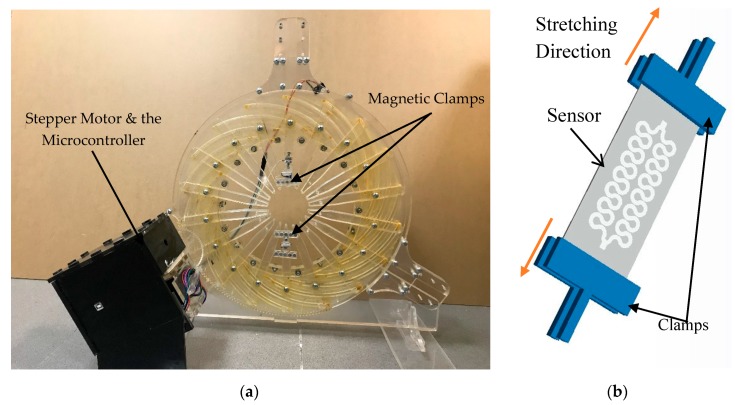
Automated stretching system: (**a**) whole setup and (**b**) sensor mounted using the magnetic clamps.

**Figure 8:**
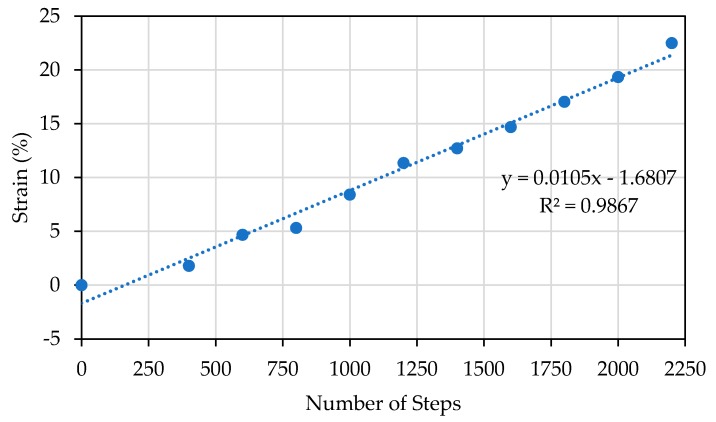
The relation between the number of steps and strain applied to the PDMS.

The selection of the optimal inkjet-printed pattern was based on the sustainability and durability tests on the automatic stretcher. As shown in Figure 7a, referring to the existing method in reference [47], a stretcher was built to apply axial and radial loads to the samples. The stretcher was automatically driven by a stepper motor whose movements were controlled by an Arduino microcontroller, based on the relationship between the number of motor’s steps and the strain for PDMS as shown in Figure 8. The sensor is mounted on the stretcher using the magnetic clamps (to reduce the stress points on the PDMS) as shown in Figure 7b where the rotational movement of the motor is converted to axial one using the gears mechanism attached to the stretcher. The stretcher was used in the comparison of mechanical properties (sustainability and durability) between different design patterns.

In the sustainability test, for a certain designed pattern, the automated stretcher applied increasing uniaxial strain on the substrates until the sensor lost its electrical conductivity. The sustainability was quantified as the maximum axial strain that the stretchable circuits can sustain without failure (resistance exceeds 1 MO hm).

In the durability test, axial cyclic strains from 2% to 5% were applied on a pattern with multiple stretching speeds (300, 500, 800 steps/s). The substrate was considered durable if it remained conductive after the cyclic durability test where the sensors were tested for an average of one hour, with a frequency of 0.7 Hz in total about 2400 cycles. Moreover, the sensitivity of the developed patterns was assessed using the gauge factor (GF) which was calculated using Equation (1).

(1) GF=(R2−R1)R1S

where $R_{2}$ is the final resistance after applying the strain (Ohm), $R_{1}$ the initial resistance (Ohm) and S the amount of strain (%).

#### 2.4.2. Repeatability and Reproducibility Tests on Respiratory Simulator

**Figure 9:**
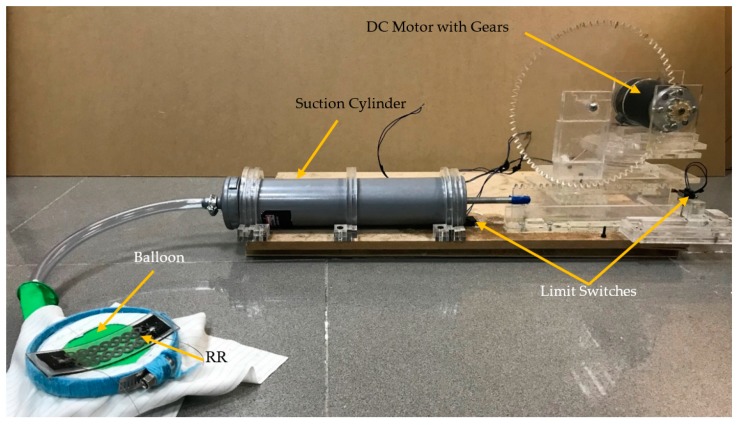
Respiration simulator prototype.

The selected pattern will be further tested for repeatability and reproducibility on the respiratory simulator. The simulator was used to mimic normal and abnormal respiratory rates to ensure the accuracy of it under different conditions. As shown in Figure 9, the respiratory simulator has an expandable surface like a balloon that mimics the movement of the human’s abdomen. The simulator consists of a 500 mL suction cylinder connected with a rack, torque magnification gear, and a pinion attached to a DC motor with an Arduino Mega microcontroller. The speed of the motor was tuned to control the respiration frequency. The simulator had two limit switches. One switch was set right next to the pump (defining maximum exhale). The other switch was next to the motor (defining maximum inhale) with position adjustments to mimic different volume strain values applied to the sensors. In addition, the simulator has a keypad and LCD to control the test and to display the simulated respiratory rate.

The volume strain ($S_{v}$) can be calculated as,

(2) $$S_{v}=\frac{\Delta V}{V_{i}}$$

where $V_{i}$ is the initial volume of the balloon container (250 mL) and Δ*V* is the change in the volume.

Table 1 lists the RR, lung volume, tidal volume, and resultant volume strain derived from adults and neonates, based on these physiological data, three respiratory modes were developed and performed on the respiratory simulator to mimic the respiratory rate of different human groups.

To test the repeatability, the IJP PDMS-based sensor was tested on two consecutive days at the same conditions on the respiratory simulator where large RR is used to mimic abnormal RRs and to inspect the mechanical properties of the sensor. The reproducibility was tested by using two sensors with the same pattern at the same conditions. The repeatability and reproducibility were investigated under the different operation modes of the simulator to confirm these characteristics in normal and abnormal RR conditions. The reproducibility reflects the consistency of results between different sensors with the performance measured at the same time which can be used to indicate the potential for commercialization. Finally, the optimal pattern was also tested on the respiratory rate simulator with different respiration frequency (motor speed 150 and 200 PWM) and different volume strain values (17.3%, 40.3%, and 59.7%) to mimic the normal and abnormal respiration of different human groups, as shown in Table 2.

### 2.5. Sensor Evaluation on Healthy Human Subjects

**Figure 10:**
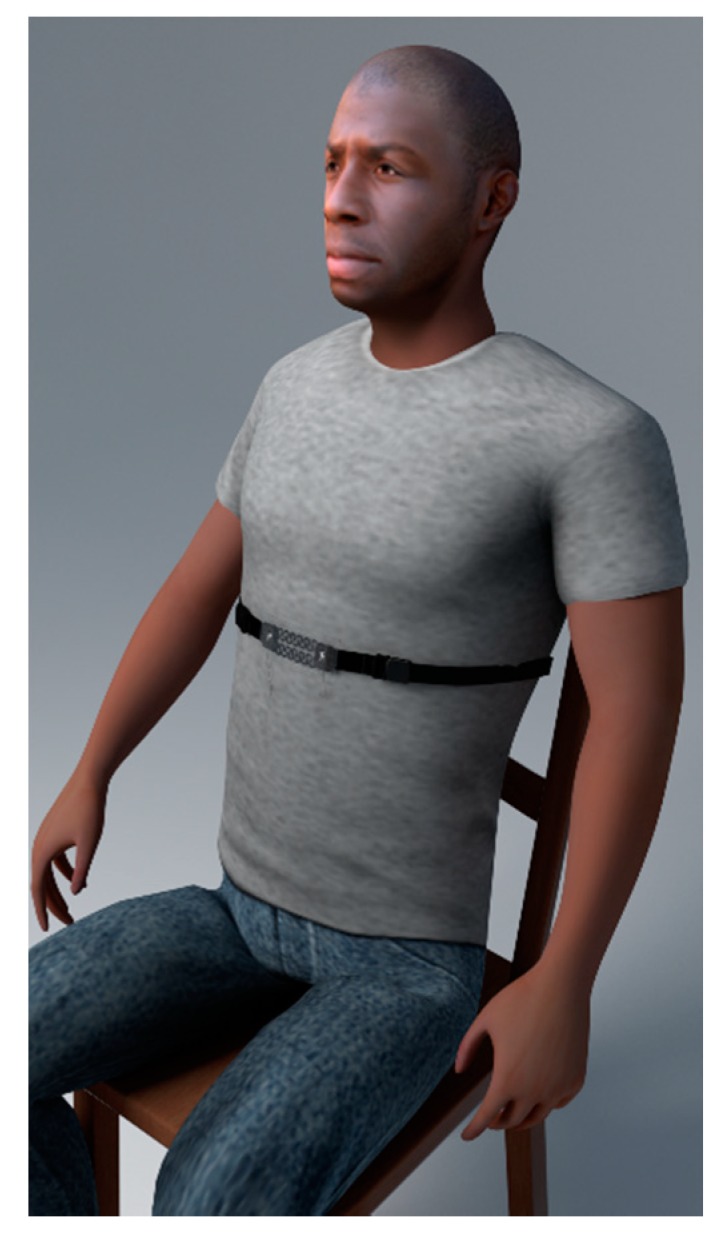
The inkjet-printed (IJP) PDMS-based sensor attached to a human at the upper part of the abdomen.

The sensor was evaluated on a group of healthy adults. The test group consisted of thirty-seven healthy subjects without active respiratory problems: thirty males and seven females with an age range of 19–34 and respiratory rate range of 11.7–31.3 BPM. The subjects were asked to take off their jackets and sit [10] on an armless wooden chair facing the computer screen in an office room where the sensor was placed at the upper part of the abdomen, as shown in Figure 10. The sensor was tested on the test subjects two times to investigate the repeatability with two minutes break between each trial [53]. An analysis Of Variance (ANOVA) test was applied to the data and used to ensure that there were no significant differences between the two measurements of each subject. The measured RR from the IJP PDMS-based sensor was compared with a previously evaluated [54,55] reference sensor (e-Health AirFlow sensor, Cooking Hacks, Zaragoza, Spain); the reference sensor is a nasal airflow sensor that senses the changes in the nasal thermal airflow and the change in the nasal air temperature [56]. The test subjects were asked to breathe normally and not to talk or move during the test [53].

## 3. Results

### 3.1. Patterns Comparison: Sustainability and Durability

The initial resistance of the evaluated patterns varies due to the variety in their geometry where design #2 and #4 had the lowest initial resistances while design #1 and #6 had the largest initial resistances. Table 3 shows the range of the initial resistance of the evaluated patterns. As for the sustainability, the maximum axial strain values for the six patterns (one to six in Figure 3) were 5%, 2%, 12%, 2%, 9%, and 7%, respectively. Patterns 2 and 4 were excluded from further durability tests since they had the lowest sustainability.

**Figure 11:**
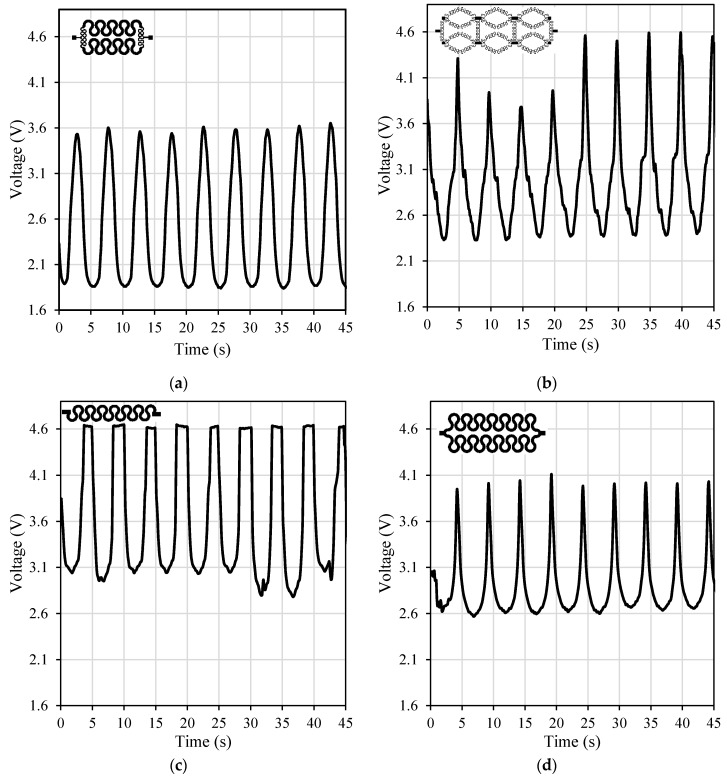
The output voltage over time from the four selected patterns under 3% axial strain at 800 steps/s speed: (**a**) design #1, (**b**) design #3, (**c**) design #5, and (**d**) design #6.

The tested patterns (1, 3, 5 and 6) had similar performance and similar output waveforms under different speeds (300, 500 and 800 steps/s) and different axial strains (2%, 3%, and 5%) where the only differences were the maximum output voltage. Figure 11 shows the performance of the tested patterns under 3% axial cyclic strain and speed of 800 steps/s. It can be observed that design #3 was more sensitive to the load with voltage values from around 2.3 volts (at zero strain) to 4.5 volts (at maximum strain, 3%) and gauge factor of 0.32 while the horseshoe pattern (design #5) was the least sensitive one with gauge factor of 0.07. Design #1 was more sensitive than design #6 with a gauge factor of 0.18 while design #6 had a gauge factor of 0.11, however only design #6 passed the durability test.

### 3.2. Sensor Testing on Respiratory Simulator: Repeatability and Reproducibility

The ability of the stretchable sensor to sustain the biggest strain under continuous operation without losing its functionality is one of the most vital characteristics of any stretchable sensor, which has to be considered carefully. As previously mentioned, the sensor with design #6 was the most durable design with breakdown strain (7%) suitable for RR measurements where it was mentioned in reference [57] that the respiration process causes a strain of ≤5%. Therefore, it was selected to be tested on the respiratory simulator at different modes. The recorded RR from IJP PDMS-based sensor was comparable with the actual values from the simulator under different volume strains and RR as shown in Table 4.

**Figure 12:**
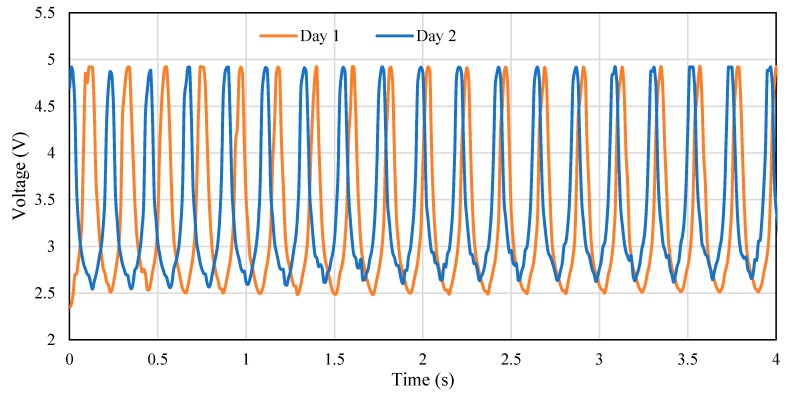
The output voltage of the sensor over time when placed on the simulator in two consecutive days under a volume strain of 17.3% and 154.2 BPM simulated by the respiratory simulator.

The RR values measured by the IJP PDMS-based sensor conformed to the RR performed on the simulator, as indicated by the small difference in Table 3 with an average error of 0.46 ± 0.66 BPM (mean ± SD). Therefore, the IJP PDMS-based sensor could accurately detect the respiratory movement and estimate RR value on the respiratory simulator. The output waveforms from the IJP PDMS-based sensor under the different operating modes of the respiratory simulator were almost identical in the two consecutive days, Figure 12 shows the performance of the sensor in two consecutive days using the respiratory rate simulator under 17.3% volume stain and 154.2 BPM. Notice in Figure 12 that the waveforms from two days are almost the same with trivial differences in amplitude (less than 2%) and period (less than 5%), which confirm the repeatability of the sensor over time.

**Figure 13:**
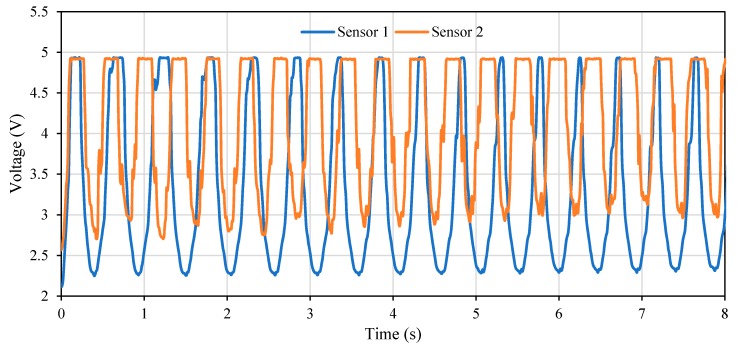
The output voltage of the two sensors with identical design over time when placed on the simulator under a volume strain of 17.3% and 58.2 BPM simulated by the respiratory simulator.

The IJP PDMS-based sensor is reproducible where the output waveforms from the two sensors were almost the same under all the test conditions and respiration modes, Figure 13 shows the results derived from two IJP PDMS-based sensors in the reproducibility test under the volume strain of 17.3% and 58.2 BPM. It can be seen that both sensors have almost the same performance where sensor one had 17 cycles, while sensor two had 19 cycles in the same time period.

### 3.3. Sensor Validation on Human Subjects

**Figure 14:**
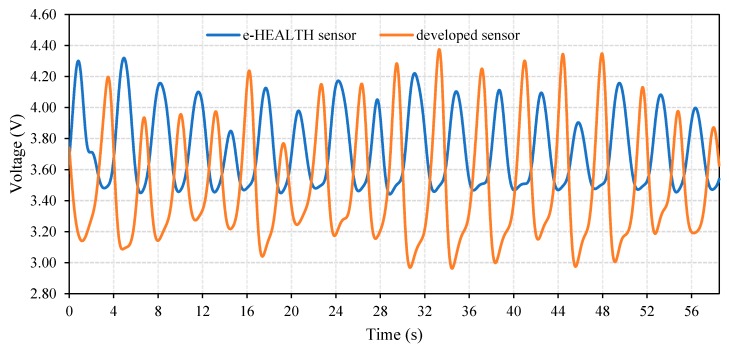
The respiratory signals derived by the developed IJP PDMS-based sensor and reference e-Health sensor. Signals were derived from subject 6 in Table A1 in the sitting position.

The respiratory signal derived from IJP PDMS-based and reference (e-Health) sensors were comparable, as in Figure 14, despite the difference in amplitude and waveform, the respiratory signals from the IJP PDMS-based sensor and the e-Health one showed identical periodical changes. It should be noted that the phase shift between the waveforms from the two sensors is due to the working principle of each sensor, which is explained in Section 4.1. Table A1 (Appendix A) shows the RR measurements from the IJP PDMS-based and reference sensors for all the test subjects. It should be noted that there was no significant statistical difference in the calculated means of the two measurement trials where the ANOVA test indicates that the *p*-value (0.221) was larger than the alpha level (0.05), and so the null hypothesis is not rejected. Finally, it can be concluded from Table A1 that the developed sensor was very accurate where there was no difference between the measured RR for the reference sensor and for the developed one.

## 4. Discussion

### 4.1. Characteristics of the Developed Sensor: Sustainability, Durability, Repeatability, and Reproducibility

The IJP PDMS-based sensor is considered as a novel low-cost stretchable and wearable sensor where the sensor was PDMS-based and was fabricated using inkjet printing technology. The sensor employs the change in the inductance of the printed pattern due to the change in the abdomen topography for the respiratory rate detection using Wheatstone bridge circuit where Fast Fourier Transform (FFT) is used to process the data obtained from the sensor. The developed sensor passed all the proposed evaluation tests, namely testing its sustainability, durability, repeatability, and reproducibility. The developed sensor can sustain axial strains up to 7% and volume strains up to 59.7% where the sensor was durable and had repeatable performance at different testing periods as shown in Figure 12. Moreover, the developed sensor was reproducible where the difference in the number of cycles reported in Figure 13 is related to the performance of the simulator where in some cases a slip between the gear and the rack occurs during the simulation which affects the output results. Moreover, the difference in the output voltage in the valleys was due to the variation in the initial resistance; the resistances of the inkjet-printed patterns vary (depending on the shape it could vary in the range of 10 to 100 Ohm) even if the same conditions were applied as the inkjet printing technology involves many variables that it was difficult to control. However, the respiratory rate detection process was not affected by the value of the output voltage.

Some WFSs have achieved accurate RR detection with zero error, but they used expensive materials and technologies such as fiber optic-based smart textile sensors as in [5] or expensive and sophisticated fabrication techniques such as photolithography [6,32] or femtosecond laser [6]. A low-cost RR sensor has been recently developed based on the RIP technique, but it had rigid islands in this sensor, which eliminated its stretchability and therefore the convenience and comfort for daily applications [2]. In another low-cost RR sensor applicable in low-resource settings, the sensor was based on a thermistor placed at the nasal outlet where it lacked wearability, stretchability and flexibility for convenient use [34]. Compared with existing works, our IJP PDMS-based sensor achieved accurate, wearable, and stretchable RR detection at a relatively low cost.

### 4.2. Accuracy of the IJP PDMS-Based Sensor

Clinically, the accurate measurement of RR needs inconvenient apparatus such as spirometer or capnometry. In this study, the accuracy of RR detection achieved by the IJP PDMS-based sensor is comparable with reference RR sensors. The measurements protocol adopted in this study is similar to the measurements protocols adopted in the literature [53] which ensures the reliability of the conducted measurements. The IJP PDMS-based sensor was accurate and competitive with the sensors reported in the [3,32,33,34,58,59,60,61] with zero BPM deviations from the actual measurements measured by e-Health nasal RR sensor when tested on healthy subjects. Moreover, the sensor was tested on the respiratory simulator over a wide range of RR to mimic normal and abnormal rates (between 23 and 154.2 BPM) where it had a small deviation from the actual RR with error of 0.46 ± 0.66 BPM (mean ± SD).

The IJP PDMS-based sensor achieved comparable or even better accuracy with the published studies. For instance, Wu et al. [2] reported a 15% relative error in the measured RR using their WFS compared with the ones measured by BioPac MP150 respiratory rate sensor. Moreover, Lee [3] reported a clinical evaluation of the use of piezoelectric sensor for RR measurements with an error of −0.41 ± 1.79 BPM and -0.58 ± 2.5 BPM compared with the electrocardiogram (ECG) derived RR and the manually observed one, respectively. Furthermore, the accuracy of the IJP PDMS-based sensor is comparable with commercial wearable sensors such as HealthPatch MD (VitalConnect, San Jose, CA, USA) where the manufacturer reported an accuracy of ±3 BPM for RR between 4 and 42 BPM [62].

In addition, the accuracy of the IJP PDMS-based sensor was comparable with or even better than the accuracy of the stretchable RR sensors in the literature [32,33]. For instance, Chung et al. [32] presented a stretchable sensor capable of measuring several vital signs including RR where they reported an error of 0.3 ± 0.95 BPM for the RR measurements while testing on adults.

### 4.3. Respiration Waveform Features

Despite the exact consistency between the RR values detected by e-Health and the IJP PDMS-based sensors, the respiratory waveforms were different (Figure 14). The e-Health sensor detects the thermal changes of the respiratory airflow. During the inhalation, there is no change in the airflow temperature. Therefore, the output voltage is constant in e-Health sensor, which could not track the changes in the inhaled airflow. By comparison, the developed IJP PDMS-based sensor detected the volumetric changes by the strain. The waveform reflects not only the fluctuations during the whole respiratory cycle of inhalation and exhalation, but also reflects the depth of breathing by the amplitude. This indicates that the developed sensor could detect RR during low and high frequency respiration, while the accuracy of the reference e-Health sensor might be affected due to the weakened airflow and thermal fluctuations.

Moreover, it can be seen from Figure 14 that the amplitude of the waveform from the IJP PDMS-based sensor varies during the test unlike that of the reference sensor, which could be related to artifacts introduced by the abdomen movement. In Figure 14, the enlarged amplitudes illustrate the deepened breathing from 30 s on. As to the relationship between the output voltage and depth of breathing on the developed sensor, the voltage depends on the strain resulted from the attachment of the belt where a pre-strain applied to the sensor could make a difference, as shown in the results derived from the simulator and the human subject in Figure 13 and Figure 14. Therefore, our IJP PDMS-based sensor could not only detect the RR value with high accuracy, but also reflect the volumetric changes, which are important for the clinical evaluation of respiration.

### 4.4. Limitations of the IJP PDMS-Based Sensor

The accuracy of the sensor is highly affected by the strain applied to the sensor where as mentioned in Section 2.1.3, the maximum strain that the sensor can sustain depends on the shape of the used pattern. For instant, overtightening the belt could break the developed sensor as the sensor can endure certain strain and if it is exceeded, the sensor will lose its conductivity and become an open circuit. The output signal from the open circuit will reach the saturation value near 5 volts, as in Figure 13. In addition, overtightening the sensor continuously could decrease the durability and the lifespan of the sensor. It should be noted that small saturations in the output signal as in Figure 13 does not affect the accuracy of the sensor where the FFT algorithm was capable of detecting these saturations as peaks. On the other hand, if the sensor was not tightened enough the amount of noise in the output signal will be higher which could affect the accuracy of the RR detection. Moreover, the thickness of the worn clothes affects significantly the accuracy of the sensor where an error of 18 BPM was reported while testing the sensor on subject #1 when a thick jacket was worn.

The developed IJP PDMS-based sensor was tested on healthy subjects while sitting only; further tests at different postures on healthy and nonhealthy subjects should be performed to ensure the compatibility of the developed sensors to meet various clinical needs. Finally, the characteristics of the developed sensor such as the stretchability, wearability and low-fabrication cost could pave the way for the use of such sensors in continuous RR monitoring in low-resources settings such as refugee camps, which would contribute significantly in enhancing the healthcare services in such settings.

## 5. Conclusions

In this study, a novel non-invasive wearable and stretchable RR sensor has been designed and fabricated using inkjet printing technology for the first time. The sensor is PDMS-based which ensures the biocompatibility, simplicity and low-fabrication cost. The sensor achieved high accuracy on human subjects with negligible error from the actual measurements recorded by the reference sensor (e-Health RR sensor). The developed sensor is therefore a promising technique to be applied in the low-source application situations and in remote settings. It detects the RR signals in a remote and user-friendly way, which makes it very suitable for many clinical practices with low user efforts and training needs.

## Figures and Tables

**Table 1:** Average lung volumes as well as the average respiration rate for adults and infants [48,49,50,51,52].

Group	Average Lung Volume (mL)	Tidal Volume (mL)	Normal Respiration Rate (BPM)	Average Volume Strain (%)
Neonates	75.4–110.2	15.6–38	30–60	14.2–50.4
Adults	4000–6000	500	12–20	8.3–12.5

**Table 2:** The specifications of the different operating modes of the respiratory simulator. PWM: Pulse Width Modulation.

Volume (mL)	Mode	Volume Strain (%)	Speed (mm/s)		RR (BPM)		Volume Flow Rate (mL/s)	
			200 PWM	150 PWM	200 PWM	150 PWM	200 PWM	150 PWM
43.3	1	17.3	101.5	52.2	154.2	58.2	219.7	113.0
100.6	2	40.3	101.5	52.2	70.2	31.8	219.7	113.0
149.4	3	59.7	101.5	52.2	51	23.46	219.7	113.0

**Table 3:** The range of the initial resistance of the evaluated patterns.

Design #	1	2	3	4	5	6
Resistance (Ohm)	18–30	10–15	15–26	8–15	19–25	30–42

**Table 4:** The measured respiratory rate by IJP PDMS-based sensor compared with the actual one using the respiratory simulator at different volume strains.

Volume Strain (%)	Actual RR (BPM)	Measured RR (BPM)	Difference (BPM)
17.3	58.2	58.2	0
	154.2	152.34	1.86
40.3	31.8	31.2	0.6
	70.2	70.2	0
59.7	23.46	23.4	0.06
	51	50.76	0.24

## Appendix A

**Table A1:** The measured and actual respiratory rate of thirty-seven adults while sitting.

#	Gender	Age	Respiratory Rate (BPM)					
			1			2		
			e-Health	Developed	Difference	e-Health	Developed	Difference
1	M	28	19.56	19.56	0	19.56	19.56	0
2	M	23	15.6	15.6	0	15.6	15.6	0
3	F	21	11.7	11.7	0	15.6	15.6	0
4	M	24	23.46	23.46	0	23.46	23.46	0
5	M	24	11.7	11.7	0	11.7	11.7	0
6	M	23	15.6	15.6	0	15.6	15.6	0
7	M	19	11.7	11.7	0	11.7	11.7	0
8	F	22	15.6	15.6	0	23.46	23.46	0
9	M	19	15.6	15.6	0	19.56	19.56	0
10	M	25	23.46	23.46	0	23.46	23.46	0
11	M	24	15.6	15.6	0	15.6	15.6	0
12	M	24	35.16	35.16	0	27.36	27.36	0
13	M	25	27.36	27.36	0	27.36	27.36	0
14	F	22	23.46	23.46	0	23.46	23.46	0
15	M	25	15.6	15.6	0	11.7	11.7	0
16	M	25	23.46	23.46	0	15.6	15.6	0
17	M	24	11.7	11.7	0	11.7	11.7	0
18	M	24	15.6	15.6	0	15.6	15.6	0
19	M	24	19.56	19.56	0	11.7	11.7	0
20	M	25	15.6	15.6	0	15.6	15.6	0
21	M	24	15.6	15.6	0	15.6	15.6	0
22	F	20	11.7	11.7	0	19.56	19.56	0
23	F	21	19.56	19.56	0	11.7	11.7	0
24	F	19	23.46	23.46	0	11.7	11.7	0
25	M	23	15.6	15.6	0	11.7	11.7	0
26	M	26	19.56	19.56	0	19.56	19.56	0
27	M	22	19.56	19.56	0	15.6	15.6	0
28	M	23	23.46	23.46	0	23.46	23.46	0
29	M	20	15.6	15.6	0	15.6	15.6	0
30	M	19	15.6	15.6	0	19.56	19.56	0
31	F	21	19.56	19.56	0	19.56	19.56	0
32	M	25	19.02	19.02	0	13.86	13.86	0
33	M	28	10.98	10.98	0	13.14	13.14	0
34	M	34	13.14	13.14	0	11.31	11.31	0
35	M	25	19.56	19.56	0	15.6	15.6	0
36	M	23	15.6	15.6	0	23.46	23.46	0
37	M	24	35.16	35.16	0	23.46	23.46	0
